# Is Educating Girls the Best Investment for South Asia? Association Between Female Education and Fertility Choices in South Asia: A Systematic Review of the Literature

**DOI:** 10.3389/fpubh.2018.00172

**Published:** 2018-07-13

**Authors:** Saba M. Sheikh, Tom Loney

**Affiliations:** ^1^Institute of Public Health, College of Medicine and Health Sciences United Arab Emirates University, Al Ain, United Arab Emirates; ^2^College of Medicine Mohammed Bin Rashid University of Medicine and Health Sciences, Dubai, United Arab Emirates

**Keywords:** contraception behavior, educational status, family planning services, female, fertility, health education, population growth, South Asia

## Abstract

**Background:** Universal education is a key strategy to enhance the well-being of individuals and improve the economic and social development of societies. A large proportion of school-aged girls in developing countries are not attending schools. Approximately one-third of South Asian girls do not attend school and in some regions only one in four girls attend primary school. Eliminating gender disparities in school attendance may lead to improvements in female education and reproductive health.

**Objectives:** To conduct a systematic review of the available data from international organizations and regional registries to explore the association between female education and fertility choices in South Asia.

**Methods:** Systematic review and synthesis of secondary data.

**Data sources:** MEDLINE, Embase, Google Scholar, World Health Organization, World Bank, United Nations Population Fund, Millennium Development Goals, Institute of Health Management, World Fact book, United States Centers for Disease Control and Prevention and regional registries were searched for papers published between 1970 and October 2016 and the included papers contained data from 1960.

**Study eligibility criteria and data abstraction process:** Studies were included if they contained data on (i) female education and/or literacy levels in South Asia; and (ii) fertility behavior in South Asian females. Quality of the included studies and extracted data were assessed by two independent reviewers.

**Results:** According to the World Bank report in 2016, the female literacy rate in South Asia has increased from 45.5% in 2000 to 57.0% in 2010 while a decreased trend of total fertility rate (i.e., number of children born per woman) was observed from 6.0 in 1960 to 2.6 in 2014.

**Limitations:** Only studies in English were included.

**Conclusion:** A negative relationship seems to exist between levels of literacy and total fertility rates in South Asian females which if further improved may contribute to longer-term improvements in maternal and child health.

## Introduction

The World Health Organization (WHO) (1) defines health as a “state of complete physical, mental, and social well-being, and not merely the absence of disease or infirmity while reproductive health, or sexual health/hygiene, addresses the reproductive processes, functions and systems at all stages of life.” The United Nations Population Fund reported that slower population growth and faster economic growth can be achieved by investments in health and family planning (2, 3). A lower fertility rate is often correlated with greater wealth, education, and urbanization (4). An emerging body of research has investigated the relationship between economic development and the decline in fertility (3, 5–7). These studies suggest that family planning programmes play a significant role in fertility decisions; however, there is widespread consensus that improvements in education may play a role in ameliorating fertility choices in females (3, 5–7).

Female education is a term which includes issues and debates surrounding all types of education including primary, secondary, tertiary, and health education for girls and women. The concept of female education includes gender equality, access to education, and poverty alleviation. Education to secondary or tertiary level appears to yield the highest return and the United Nations population fund report (8) identified links between “education and higher wages, increased national income, smaller more sustainable families, reduced inequalities, and a reduction in severe poverty.” Moreover, improving female education may help to eliminate child, early, and forced marriage; thus, decreasing the total number but increasing the individual health of children within a family. Compared to lesser educated women, highly educated women tend to marry later, have a higher age at first birth, and fewer children (8, 9). For example, a study in Italy reported a significant negative correlation between a female's education and her number of children (9). Child survival has been found to improve with the level of maternal education and better survival rates are usually seen in the offspring of mothers with only primary school education compared to the offspring of mothers with no education (10). Each additional year of education has been reported to lead to a 7–9% reduction in mortality for children under 5 years of age (11). This observation of falling infant and child mortality rates is evident in many developing countries who are undergoing a demographic transition of increasing female educational attainment (12).

South Asia (i.e., Afghanistan, Bangladesh, Bhutan, Maldives, Nepal, India, Pakistan, and Sri Lanka) with an estimated population of 1.846 billion in 2016, is one of the most densely populated geographical regions in the world (13). The low status of women in South Asian countries may be an important determinant of the high prevalence of reproductive health issues in the region. Total fertility rate (TFR) is the theoretical total number of children born or likely to be born to a woman in her lifetime if she were subject to the prevailing rates of age-specific fertility in the population. Theoretically, increased educational levels among females may lead to improved fertility choices and a decline in TFR. Overall, this may lead to improvements in the health and status of women, in addition to potentially providing additional social and economic benefits for their families and communities (13). The primary aim of this systematic review was to explore the association between female education and fertility choices in South Asia.

## Materials and methods

This paper has been written in accordance with the Preferred Reporting Items for Systematic reviews and Meta-Analyses (PRISMA) guidelines (14).

### Eligibility criteria

Only empirical research papers and/or official websites of organizations containing data on (i) female education and/or literacy levels in South Asia; and (ii) fertility behavior in South Asian females were eligible.

### Exclusion criteria

Articles that were not available in English and duplicate publications or sub-studies of included articles were excluded.

### Information sources

The methods used to obtain secondary data from existing peer-reviewed journal articles and related health organizations were: (1) systematic electronic searches of several databases using MeSH and keyword search terms; and (2) electronic and manual searches of publications/reports of international health authorities and governmental agencies.

### Search

The literature search was conducted from 1970 to October 2016 in PubMed, Embase, and Google Scholar using a combination of MeSH terms, free-text words, and entry terms such as “Contraception Behavior; Educational Status; Family Planning Services; Female; Fertility; Health Education; Population Growth; South Asia.” The literature search was conducted in accordance with the PRISMA guidelines. A manual search of the reference list from eligible articles was conducted to identify additional relevant articles. Electronic and manual searches of online databases and publications/reports from international health authorities and governmental agencies were also conducted.

### Study selection

The titles and abstracts of studies returned by the electronic search were initially reviewed for eligibility and the full-text of studies fulfilling the inclusion criteria were retrieved for a more detailed review. If there was uncertainty regarding the relevance of any article, the full-text version of the paper was assessed to determine whether it was included in the literature review. All studies were retrieved from PubMed, Embase, or Google Scholar database. The search period ranged from 1970 to October 2016 and the included papers contained data from 1960.

## Results

**Figure 1:**
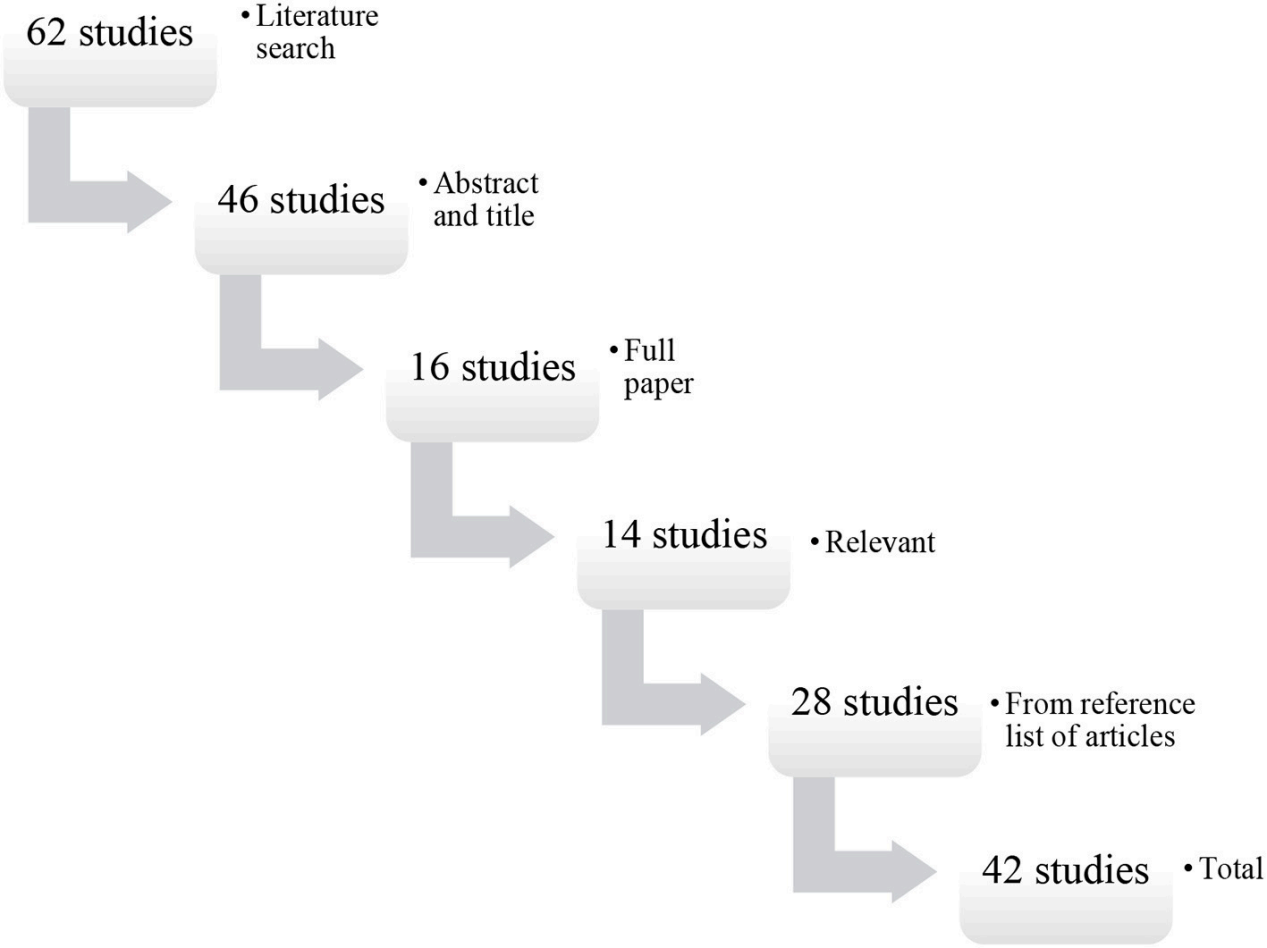
Flow diagram of the search and selection process.

The literature search returned 62 articles and the abstracts of 46 studies were reviewed. Full-text versions of 16 studies were reviewed and 14 satisfied the eligibility criteria. Sixteen articles were excluded during the initial screening of titles as they were not related to South Asia, 30 studies were excluded during the abstract review phase, and two studies during full-text review as they did not contain data on female education and/or literacy levels, and/or fertility behavior in South Asian females. An additional 28 eligible studies were retrieved from the reference lists of articles using the same exclusion criteria described above. A total of 42 eligible studies were included in this systematic review not including additional data from international organizations and regional registries (Figure 1). The 42 eligible studies included seven papers on India, five on Nepal, three on Bangladesh, three on Pakistan, two on Sri Lanka, and 22 papers that contained data on South Asia as a region and/or a range of South Asian countries.

### Globally

Since 1990, progress has been seen in female educational attainment in countries across Latin America, the Middle East and North Africa, Sub-Saharan Africa, and South Asia. Compared to developed countries, the average number of educational years for people in the developing world is lower. However, the rate of increased educational attainment is high with many countries doubling their average years of education over the past four decades. Globally, the average number of educational years has increased by 35% to 12.9 years for women and by 30% to 13.1 years for men between 1990 and 2014 (15). For those aged 25 years and older in developing countries, the mean years of education improved from 3.4 to 8.0 years in men and from 1.9 to 6.0 years among women between 1970 and 2014 (15).

An increase in primary education enrolment in developing countries was observed from 83% in 2000 to 91% in 2015; however, 57 million children of primary school age were reported to be out of school in 2015 (16). In addition, an improved global literacy rate from 83% in 1990 to 91% in 2015 has also been reported among youths aged 15–24 years. Moreover, there has been a narrowing of the gap in the net enrollment ratio for primary education between boys and girls in two thirds of developing countries (16).

### South Asia

**Figure 2:**
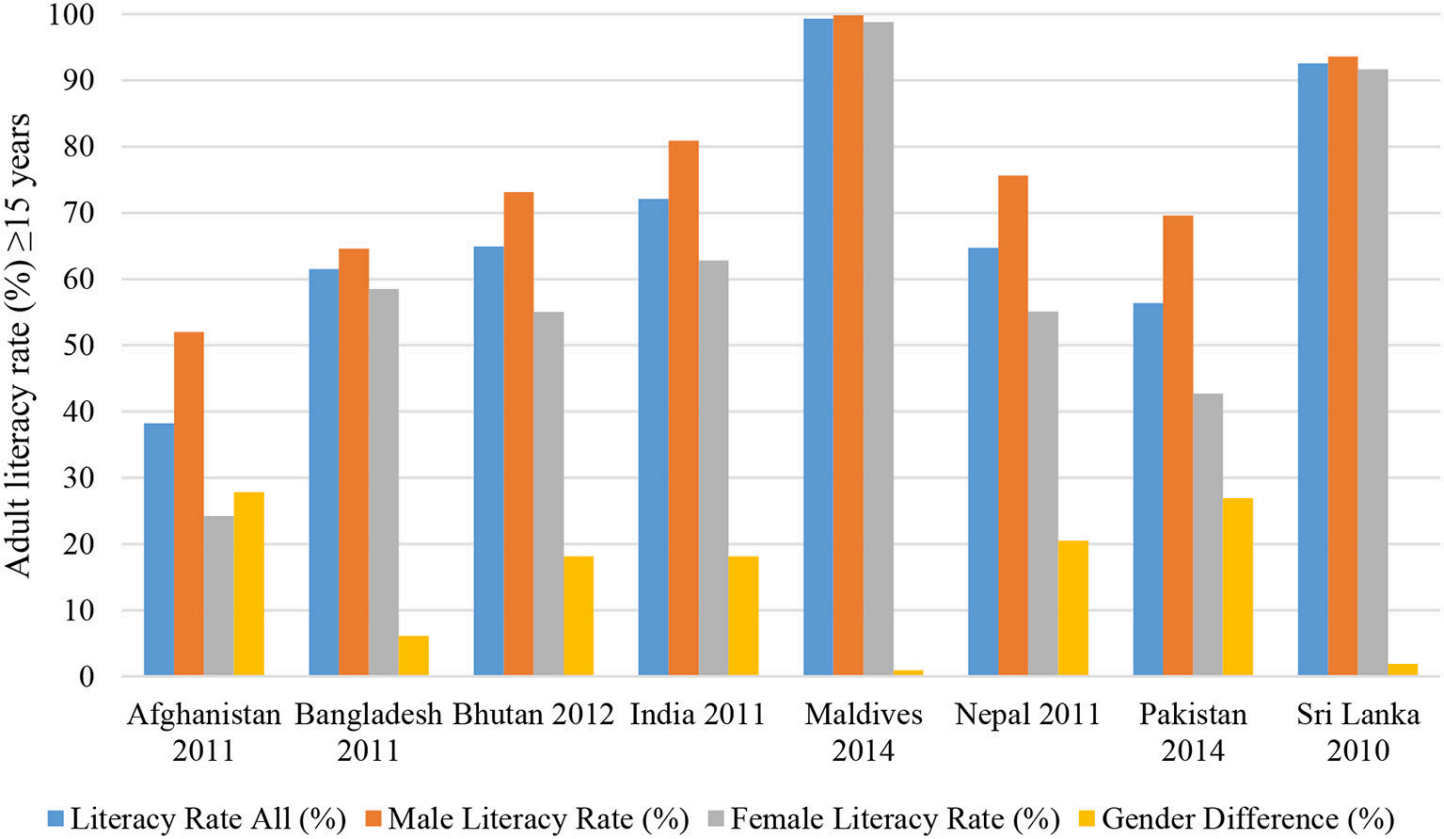
Adult literacy rate ≥15 years (both sexes, female, male) in South Asian countries 2010–2014 (11, 13).

Overall, adult and female literacy rates are quite varied across South Asian countries with the Maldives and Sri Lanka showing the highest rates of male and female literacy rates (both combined and separately) and Afghanistan, Bhutan, Nepal, and Pakistan with the lowest female literacy rates and greater gender differences (Figure 2). The overall adult literacy rates for South Asia have increased from 45.5% in 2000 to 57.0% in 2010 (13, 17). The enrollment of girls in primary schools in South Asia was only 74 girls for every 100 boys in 1990 but by 2012 the enrolment ratios have become the same for girls and boys (16).

**Figure 3:**
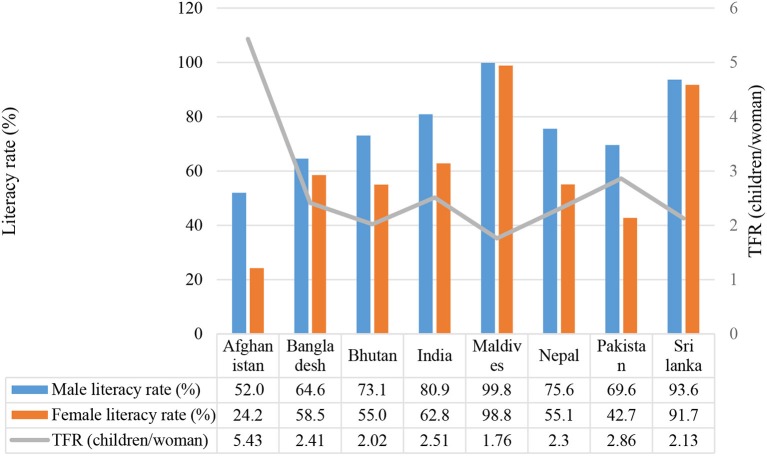
Female literacy and total fertility rates in South Asian countries [Source: (1) Adult literacy rate male and female 15+years, UIS Data center. UNESCO August 2015; (2) TFR, Total Fertility Rate (children/woman) World Health Organization (35)].

Globally, there seems to be a positive association between female education and age at marriage which usually, but not always, results in a decreased TFR and a reduced family size. According to the World Bank report there has been a decline in global TFR from 5.0 in 1960 to 2.5 in 2014 (18). A similar trend has been observed in the developing world, and the TFR for South Asia has decreased from 6.0 in 1960 to 2.6 in 2014 (18). Although there is usually a delay between an increase in female literacy rates and consequential declines in TFR, Figure 3 shows an emerging trend for countries with female literacy rates similar to male literacy rates to have a lower TFR compared to countries where the female literacy rate is lower than the male literacy rate. For example, the female literacy rate (24.2%) in Afghanistan is half the male literacy rate (52.0%) and the TFR is 5.43; whereas the male and female literacy rate in the Maldives is 99.8% and 98.8% (respectively) and the TFR is 1.76. A decline in TFR may have a long term effect as a positive correlation seems to exist between parental TFR and offspring TFR; specifically, that offspring tend to have a similar number of children as their parents (4, 5). Some demographic forecasters predict that after adjustment for gender imbalances, the effective global TFR will fall below replacement rate (i.e., < 2.1) between 2020 and 2030 (19). This may lead to stability in World population by 2050, which is sooner than expected by the UN Population Division (19).

Poverty, illiteracy, and social inequity are some of the factors related to high rates of poor maternal and child health in South Asia (20). Recent evidence suggests that a reduction in maternal mortality ratio (i.e., no. of maternal deaths per 100,000 live births during a specific time-period) can be achieved by improving the status of women in society, increasing female literacy rates, and investing in women's health (20). In South Asia, a decline in maternal mortality ratio seems to be related to the level of maternal education and this may be due to educated mothers having less children and more optimal inter-pregnancy intervals. Table 1 shows a trend for South Asian countries with higher levels of female literacy to have a lower maternal mortality ratio. For example, Sri Lanka has a female literacy rate of 92% and a maternal mortality ratio of 30 deaths per 100,000 live births compared to Nepal which has a female literacy rate of 55% and a maternal mortality ratio of 258 deaths per 100,000 live births. In addition, an increase in the mean number of years of education may have contributed to the observed reduction in child mortality. Between 1970 and 2009 an estimated 4.2 million (51.2%) of 8.2 million fewer deaths in children younger than 5 years were attributed to improved educational attainment of women in their reproductive years (21).

**Table 1:** Gross domestic product (GDP; international dollars), female literacy rate (%), and maternal mortality ratio (MMR; per 100,000 live births) for South Asian countries, 2015 [Source (1) World Bank (1, 36), (2) United Nations (18), and (3) UNESCO Institute for Statistics (22)].

	**GDP (Int.$)**	**Female literacy rate (%)**	**Maternal mortality ratio (per 100,000 live births)**
Afghanistan	1,933	24	396
Bhutan	7,816	55	148
Bangladesh	3,123	59	176
Maldives	12,637	99	68
India	6,020	63	174
Nepal	2,374	55	258
Pakistan	4,811	43	178
Sri Lanka	11,739	92	30

A high maternal and child mortality persists in most South Asian countries despite their economic improvement. However, Sri Lanka recorded the lowest maternal mortality ratio in 2015 among South Asian countries and this was partly due to sustained investments in improving primary health care and providing universal education across the Sri Lankan population (Table 1). Contraception plays an important role in controlling TFR. Two commonly used indicators are contraceptive prevalence and unmet need for family planning. Contraceptive prevalence is the percentage of women who are currently using, or whose sexual partner is currently using, at least one method of contraception, regardless of the method used (usually reported for married or in-union women aged 15–49 years) (23). The “in-union” group includes women living with their partner in the same household and who are not married according to the marriage laws or customs of a country. Unmet need for family planning is defined as the proportion of women of reproductive age with unmet contraception needs who are fecund and sexually active but are not using any method of contraception, and report not wanting any more children or wanting to delay the next child (24).

**Figure 4:**
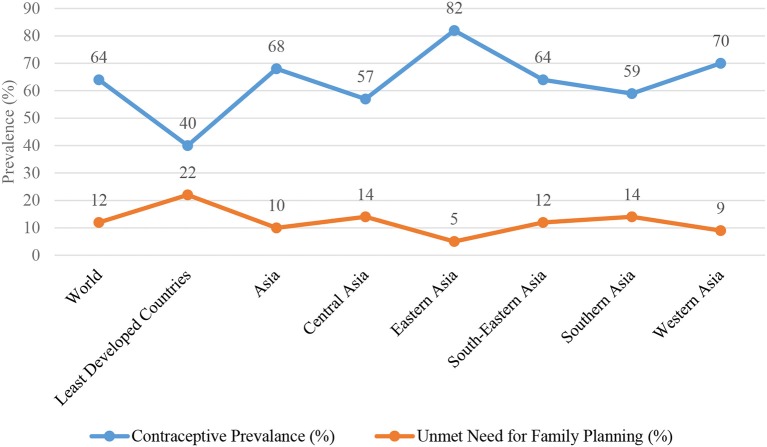
Contraceptive prevalance and unmet need for family planning among married or in-union women aged 15–49 years, 2015 [Source: United Nations (25)].

Within Asian sub-regions, Eastern Asia has the highest (82%) and Central Asia (57%) and South Asia (59%) have the lowest contraceptive prevalence (Figure 4). In 2015, China had an estimated 83% contraceptive prevalence while levels of 50% or more were estimated in 37 of the 48 countries and 70% in 10 countries of Asia. The contraceptive prevalence reported in Afghanistan and Timor-Leste was 29% which was the lowest in the region (25).

## Discussion

Reproductive health is a crucial part of general health and human development as it influences the broader context of people's lives including their living conditions, family environment, social/gender relationships, economic circumstances, employment, and education. Childhood health conditions are reflected in the future reproductive health status of the population and can affect health beyond the reproductive years for both women and men. Moreover, parental reproductive health may affect the health of the next generation as the health of a newborn is largely a function of the mother's health, nutritional status, and access to health care (26). Thus, increasing female educational levels along with serving the aim of human rights and social justice may also play a role in determining family size (9, 12, 19). Educated women generally have higher health literacy and make better use of reproductive health and family planning services to achieve their desired family size (12, 20). Educational opportunities for girls and women should not only include general and professional education but also health, literary and non-literary education which will help to empower them to take control over their own lives, health, and fertility.

In South Asia, the South Asian Association for Regional Cooperation (SAARC) was established in 1985. It is an economic cooperation organization in the region and includes all eight nations of South Asia (Afghanistan, Bhutan, Bangladesh, Maldives, Nepal, India, Pakistan, and Sri Lanka) which apart from geography share similar economic and health issues. According to the report for Selected Countries and Subjects (27) in 2015, 3% of the world's area is composed of SAARC countries, 21% of the world's population lives there, and it constitutes 9% of the global economy. One of the major challenges faced by South Asia is the high number of out-of-school children. Despite serious efforts toward education, household surveys by UNESCO (22) showed that in Bangladesh, India, Pakistan and Sri Lanka a total of 27 million children between the ages of 5–13 years were out of school, of which 17 million children were of primary school-age (aged 5–9 years in Pakistan and Sri Lanka, and aged 6–10 years in Bangladesh and India). Moreover, 9.9 million children were of lower secondary school-age (aged 10–12 years in Pakistan, 10–13 years in Sri Lanka, and 11–13 years in Bangladesh and India) and the majority of children in each of the three largest countries lived in rural areas. Initiatives including school fee abolition and community-based efforts have helped to improve enrolment rates which increased from 75% in 2000 to 89% in 2011. However, there is still a need for enhanced and fully implemented policies and programs to overcome the socio-cultural barriers in the region such as the practice of early marriages and child labor. In addition, there is a need for reforms related to improving the quality of education at classroom level, especially in the rural and other marginalized areas which have a higher proportion of out-of-school children. In the South Asian region, female education has faced significant obstacles in Afghanistan (28) where it was reported that the majority of girls' schools were closed by the Taliban. According to Education for All, the gross enrollment ratio for girls in Kabul in 1995 prior to the Taliban take over was 32% which had fallen drastically to 6% in 1999 (29). After the fall of the Taliban, the Afghan government made education one of its top priorities and the government has been facilitated in its efforts by donors in ensuring children attend school, particularly at primary level.

Progress is also observed in the region with increasing primary school enrolments and falling gender gaps; (30–32) however, many children leave school prematurely without finishing their education. Challenges persist in increasing enrolments and decreasing dropout rates particularly for girls belonging to poor families and living in the remote areas or culturally conservative settings. It is noted that in the South Asian region, the enrolment of girls in secondary schools remain generally low; however, it has been anticipated that improving this could pay off tremendously in South Asia. Increasing female education has been reported to have helped slow population growth, raise productivity of the present and future generations, reduce the education gender gap, and promote faster growth of per capita income (33, 34). Taking all these benefits into account, economists have calculated that female education may well be the highest-return investment available in developing countries today.

### Female education and total fertility rate (TFR)

Total fertility rate (35) refers to births per woman and is a more direct estimate of the level of fertility than crude birth rates. A rate of 2.1 children per woman is considered the replacement rate for a population which can result in relative stability in terms of total numbers. However, rates >2.1 children indicate increasing population size with decreasing median age and in some situations may lead to difficulties for families in feeding and educating their children. On the contrary, a TFR of < 2.1 indicates a population which is decreasing in size and will eventually lead to an older population structure (i.e., greater proportion of older people in the population). Increasing female education changes the dynamics of family formation as it shortens the total reproductive life of a woman leading to a decreased TFR, population growth, infant, child, and maternal mortality, and improvements in overall family health and wealth (12, 26). In societies where childbearing is socially unacceptable prior to marriage, female education plays a significant role in influencing the timing of marriage, contraceptive use, and age at first conception for women (12, 23). The World Bank (18) has reported that the global TFR has steadily declined from 5.0 in the 1960s to 2.5 in 2014 with a similar trend noted in developing countries. This change was found to be most pronounced in industrialized countries, especially Western Europe, where over the next 50 years the population growth is projected to decline (36). In South Asia, the TFR has declined from 6.0 in 1960 to 2.6 in 2014.

More than three decades ago, Kasarda (37) observed a link between female education and fertility but felt the need for systematic empirical tests to assess the role of education on lowering fertility at individual or aggregate levels. Kasarda (37) stressed the need for a refined conceptualization of the education-fertility relationship, in order to discover different aspects of female education which may play a role in reducing fertility and also to identify the causal variables and operators that mediate the effects of female education. A study in Pakistan by Sathar and Mason (38) identified four significant intervening variables in the education-fertility relationship; namely, marriage duration, net family income, formal sector employment, and age at first marriage. Moreover, if these intervening variables are kept constant then the attitude of women towards family planning loses its impact on fertility. In view of these findings, there is a need for increased investments in female education as this will indirectly lead to increased human and economic development. One of the limitations of the present review was the lack of available data on possible intervening variables that might influence the education-fertility relationship. Future studies investigating the relationship between female education and fertility should plan to include data on all possible intervening and moderator variables. Such data would allow a more empirical analysis of the proposed relationship between female education and fertility. The major tenet of this review is that there seems to be an inverse relationship between female education levels and fertility rates; specifically, that an increase in female education levels leads to a reduction in TFR at both the individual and population level. That said, reverse causality is also plausible whereby females decide to have few children in order to continue their education. As the present review utilized secondary cross-sectional data, it is not possible to draw firm conclusions about the temporal or causal nature of the association between female education and fertility.

A study in India by Borkotok and Unisa (39) found that female educational levels may influence age at marriage with 87% of uneducated women married by the age of 20 years compared to 73%, 59%, and 17% of women with primary, high school, and higher education, respectively. Moreover, this study found that delaying marriage due to higher female educational attainment increased the possibility of a woman marrying a man with an equal or higher educational status than themselves which in the long-term has been found to contribute to decreased school drop-out rates. Similarly, a study in Nepal (40) used a household-level dataset to examine the relationship between socioeconomic characteristics, age at marriage, and TFR. The study reported that the relationship between age at marriage was more strongly associated with female educational levels compared to male educational levels. Data from the Demographic and Health Surveys for 26 countries (41) also confirmed these results and showed that higher education is consistently associated with lower fertility even at the lower end of the educational range in some of the least-developed countries. This study also examined the impact of female education on age at marriage, family-size preference, and contraceptive use. The study findings support the view that education may enhance a woman's ability to make healthy and optimal reproductive choices. In Bangladesh, fertility and mortality rates declined only slightly during the 1970s and the population continued to grow at an annual rate of ~2.5%, implying a doubling time of ~25–30 years (41). The study found that nearly all women in Bangladesh were married by the age of 25 years and that more educated women married later than less educated females (41). In 1982, the United States Agency for International Development began funding a pilot project by the Bangladesh Association for Community Education to provide secondary school scholarships for girls in Chandpur District. Only 30% of secondary school completers had married by the time of the survey, in comparison to 76% of secondary school dropouts, 77% of the primary school completers, and 66% of those with no formal schooling (42).

In South Asian countries, families and traditions influence the educational attainment of women. Data from a 2002 survey in six villages in rural Bangladesh (43) observed an association between mother and mother-in-law's education and the next generation's decision on the timing of marriage and childbearing. In Nepal, traditionally most household decisions are made by the senior male member, and in such families there is a trend of having more children compared to families where women have more influence on household decisions including fertility choices (44). In the South Asian region, Sri Lanka has nearly traversed the demographic transition (i.e., shift from high birth and death rates to lower birth and death rates as a country/region develops) and now has low mortality and fertility rates with the latter reaching replacement fertility levels (i.e., TFR < 2.1). A possible explanation for these differences is that compared to northern South Asia where relations between son and parents are central to the family structure, in a Sri Lankan family the position of women is relatively high from a South Asian perspective and consequently women have the autonomy to make choices related to their health and fertility behavior (12, 45). In summary, future government policies in South Asia should consider focusing on enhancing current and developing new female education programs to increase female literacy rates and improve reproductive health.

### Female education and contraception

Female education has direct and indirect effects on fertility by affecting reproductive choices including breast feeding patterns and contraceptive use which both have the potential to reduce the gap between actual and desired family size. The medical benefits of contraceptives, maternal health services, and services related to sexually transmitted infections include preventing high-risk pregnancies, obstetric complications, unsafe abortions, and reducing cancers of the reproductive system and deaths related to the human immunodeficiency virus. At a community level, these services contribute to improving the nutritional status of women and their children which decreases the risk of anemia and infertility for women, and improves the health and survival rate of infants. Proper education about access to safe, effective, affordable and acceptable methods of family planning for fertility regulation should be provided to all men and women. In addition, appropriate health care services should be provided to all women to enable them to have a safe pregnancy and childbirth which will maximize their chance of having a healthy infant.

The landmark program of action of the International Conference on Population and Development in 1994 recommended that all countries provided universal access to a full range of safe and reliable family-planning methods by the year 2015 (46). Since then, there has been increased investment in family planning programs in many developing countries and this has led to reduced fertility rates by improving knowledge about birth control and access to contraceptives (7, 39, 47). In India, low levels of female education are partially responsible for impeding the efforts implemented for improving the population's quality of life by increasing female accessibility to family planning methods (47). Sarkar (47) found that rural women experienced 0.8 more live births than their urban counterparts and this observation may be partly due to the higher education of women living in urban areas. Indeed, female education has been shown to be a strong determinant of lowered fertility and this may be due to the effect of education increasing age at first marriage and the adoption of modern contraceptive methods (18). Effective contraceptive use is still impeded by a lack of understanding related to reproductive physiology and existing prejudices and taboos. Nonetheless, the importance of education in general is undeniable as educated women may be more likely to discuss family regulating plans with their husbands (39). A positive association exists between the educational levels of males and his favor of female education, contraceptive use, and family size (48). A recent study in Pakistan found that among uneducated males 32.7% did not discuss any family planning methods with their wives compared to 11.3% of educated males (*p* < 0.001) (48).

Improved access to contraceptives is necessary to reduce fertility rates, especially in countries which traditionally favor a large family. Socio-cultural and socio-economic development will be stalled in these areas until measures are implemented to increase the availability of family planning services (49, 50). Research in the South Asian region has suggested that the low autonomy of women and the strong preference for sons are major impediments to sustained declines in TFR and the adoption of safe and modern contraception (51). In some areas of South and East Asia where there is a preference for sons, the decline in fertility rates has led to a noticeable gender bias in the population (51). For example, in some parts of India couples are resorting to female feticide or infanticide in order to achieve their desired family size and sex composition of children (52–54). Morgan et al. (55) noted that religion emerges as an important determinant of the preference for sons and that in comparison to Hindus, Christians and Muslims had a lower preference for sons but found that Sikhs had a stronger preference for sons than Hindus. Some religious bodies have passed strictures against the practice of female feticide in their communities; however, increasing female education and consequently economic status may help to increase the value of daughters and reduce the preference for sons.

## Future directions

Millennium Development Goal 5 (25) was to improve maternal health. Despite some progress, the global effort to reduce maternal deaths and improve universal access to reproductive health fell short of the targets set to be achieved by 2015. The Global Strategy (2016–2030) “Every Woman Every Child Movement” (56) is a platform for accelerating the health-related Millennium Development Goals and it has brought women, children, and adolescents at the center of the new UN Sustainable Development Goals. The strategy recognizes that in order to achieve sustainable development, it is necessary to create harmony in three core elements including economic growth, social inclusion, and environmental protection. All these elements are interconnected and are crucial for the well-being of individuals and societies.

Some of the poorest countries in the South Asian region have experienced an increase in the level of female educational attainment over the past few decades. While this progress is encouraging, there is a need for governments to prioritize female and male education to provide sufficient financial, managerial, and manpower resources in the future. Community centers, religious institutions, and the broader civil society can also be mobilized to facilitate the education of all children. In addition to increasing educational enrolment among children, there is a need to improve the quality of the education that is delivered. Traditionally, schools in South Asia have employed a rote-learning approach to education and this needs to shift to a child-centered problem-solving approach. Curriculums may require a thorough review to ensure that they are equipping children with the skills that will be required in the future employment market of these developing countries, many of which have experienced rapid economic growth over the past two decades.

## Limitations

Our search strategy involved conducting independent searches on three different electronic databases (i.e., PubMed, Embase, and Google Scholar) for papers published in English. In addition, we performed electronic and manual searches of publications/reports of international health authorities and governmental agencies. Despite these efforts, it is possible that papers meeting our inclusion criteria published in other languages and/or indexed on different databases were missed and therefore, not included in the review. Finally, the studies included in the present review did not contain data on possible intervening variables (e.g., marriage duration, net family income, formal sector employment, age at first marriage) that might influence the education-fertility relationship.

Our study utilized available secondary data from eight low- and middle-income developing countries in South Asia to explore the relationship between female education levels and fertility choices in South Asian females. While data pertaining to fertility choices were available from 1960 to 2014, data on levels of female education were only available from 1990–2015 for global estimates and 2000–2015 for South Asia. Total and gender-stratified enrolment ratios and completion rates for primary, secondary, and tertiary education are important indicators that should be collected in annual surveys. In addition, male and female adult literacy rates are useful indicators to track the outcomes of primary and secondary education programs. Periodic and longitudinal monitoring of these educational indicators along with fertility data (e.g., TFR, contraception prevalence) would facilitate future studies to further explore the association between female education levels and fertility choices. Moreover, future longitudinal/prospective cohort studies collecting educational data at various stages of schooling and following the participants throughout their reproductive life course would determine the temporality, direction, and strength of association between female education levels and fertility choices.

## Conclusion

Empowerment of women through education seems to play an important role in improving reproductive health practices. Consequently, this may help to reduce the gender gap and enhance the health, status, and roles of women in society. Our study suggests the possibility of a negative relationship between female education level and TFRs in South Asian females. The main tenet is that a rise in female education rates at the population level will contribute to a decline in fertility, population growth, and infant and child mortality. Evidence from South Asia suggests that improving female education may have significant health, economic, and social benefits for families, communities, and countries. South Asian countries should further develop and expand their educational programs for girls and boys with the primary aim of achieving the highest attainable standard of health and wellbeing for all women, men, adolescents, and children. South Asia consists of low- and middle-income countries that may lack the sufficient resources to achieve full coverage of education for all school-aged females and males. Therefore, these countries may require greater developmental assistance and funding from external organizations to implement innovative and cost-effective pedagogical strategies, such as tele-education, to increase education levels among school-aged children.

## Author contributions

SS conceived the idea and TL supervised the project. SS drafted the manuscript and TL revised the manuscript. Both TL and SS critically reviewed, provided intellectual input to the manuscript and approved the final version of the manuscript.

### Conflict of interest statement

The authors declare that the research was conducted in the absence of any commercial or financial relationships that could be construed as a potential conflict of interest.
